# Supplementary material for: Eukaryotic Translation Initiation Factor 4AI: A Potential Novel Target in Neuroblastoma
Source: Cells. 2021 Feb 2;10(2):301. doi: 10.3390/cells10020301 (PMC7912938; doi:10.3390/cells10020301)
Supplement: Supplementary file 1 [file cells-10-00301-s001.zip › Supplementary_cells-1052933/Supplementary_Table_1_cells-1052933.docx]

Supplementary Table 1: Clinicopathological data of patients analyzed in the Therapeutically Applicable Research to Generate Effective Treatments (TARGET) initiative.

| **Age at Onset (Mean±SD)** | | 3.5 ± 3 months |
| --- | --- | --- |
| **Gender** | |  |
|  | **Female** | 59 (42%) |
|  | **Male** | 83 (58%) |
| **Stage** | |  |
|  | **3** | 6 (4%) |
|  | **4** | 116 (82%) |
|  | **4S** | 20 (14%) |
| **MYCN-Status** | |  |
|  | **Not Amplified** | 111 (78%) |
|  | **Amplified** | 30 (21%) |
|  | **Unknown** | 1 (1%) |
| **Localization** | |  |
|  | **Adrenal** | 84 (59%) |
|  | **Non Adrenal** | 58 (41%) |
