# Supplementary material for: Eukaryotic Translation Initiation Factor 4AI: A Potential Novel Target in Neuroblastoma
Source: Cells. 2021 Feb 2;10(2):301. doi: 10.3390/cells10020301 (PMC7912938; doi:10.3390/cells10020301)
Supplement: Supplementary file 1 [file cells-10-00301-s001.zip › Supplementary_cells-1052933/SupplementaryFigure1Caption_cells-1052933.docx]

**Supplementary Figure 1:** **Immunohistochemical** **Protein Expression Analysis of eIF4E in Neuroblastic (NB) and Non-neoplastic Tissue (NNT).**
(**A**) Representative immunohistochemical staining with no, weak and moderate eIF4E immunoreactivity of NB tissue and no staining of NNT. Scale bars: 200 µm for upper row and 50 µm for bottom row.
(**B**) Comparison of immunohistochemical eIF4E staining intensity of NB (n=36) and NNT (n=18) using Mann-Whitney *U* test. *** p<0.001; no staining (white bar) NB n=19, NNT n=18; weak staining (light grey bar) NB n=14; moderate staining (dark grey bar) NB n=3.
(**C**) Kaplan-Meier curves based on high versus low *EIF4A1* expression, MYCN amplification status (amplified versus non-amplified) and localization (adrenal versus non-adrenal). RNASeq data of 141 NB patients generated within the Therapeutically Applicable Research to Generate Effective Treatments initiative was analyzed using R version 4.0.2.
