# Supplementary material for: Eukaryotic Translation Initiation Factor 4AI: A Potential Novel Target in Neuroblastoma
Source: Cells. 2021 Feb 2;10(2):301. doi: 10.3390/cells10020301 (PMC7912938; doi:10.3390/cells10020301)
Supplement: Supplementary file 1 [file cells-10-00301-s001.zip › Supplementary_cells-1052933/SupplementaryFigure2_cells-1052933.pdf]

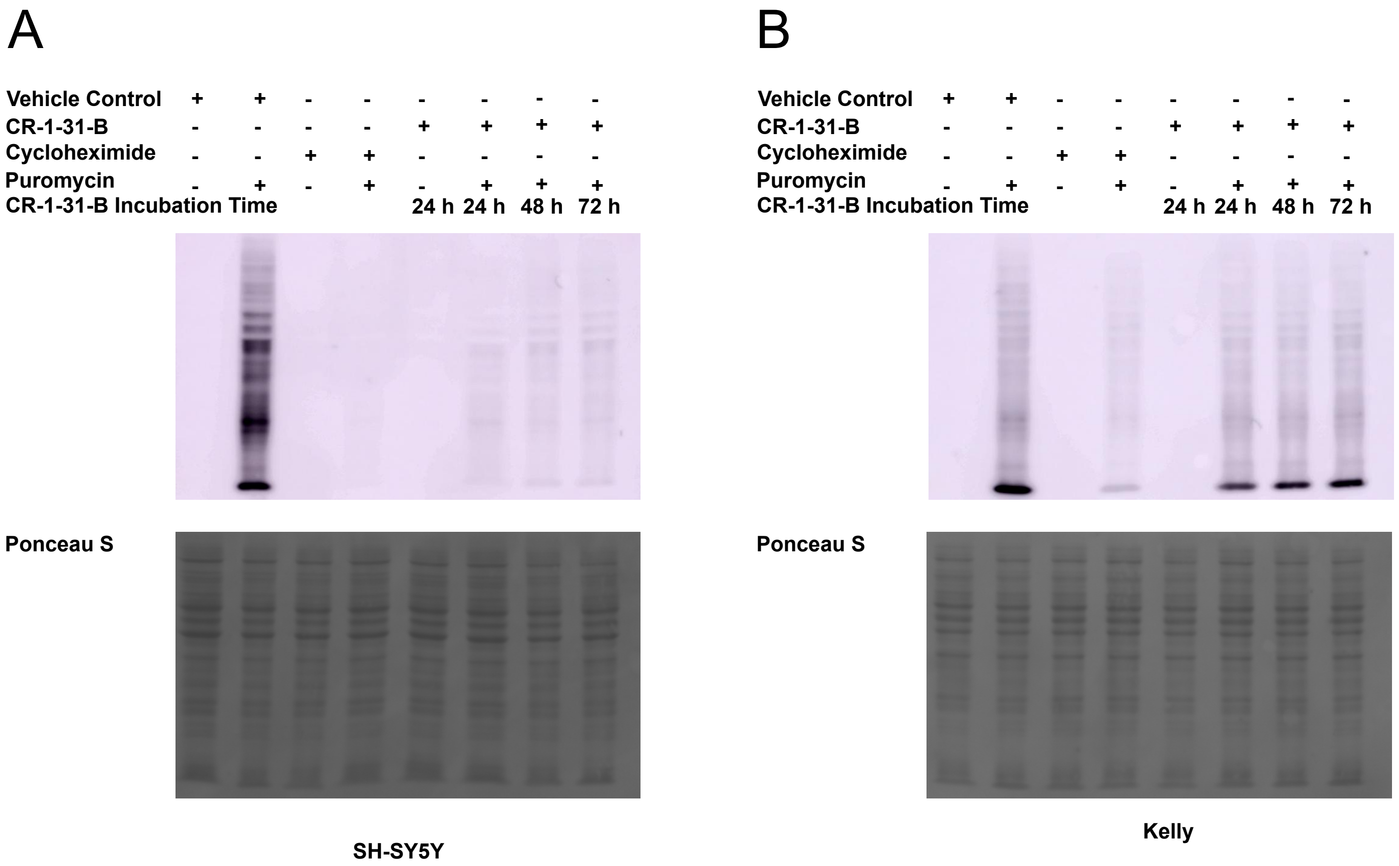
A
Vehicle Control + + - - - - - -
CR-1-31-B - - - - + + + +
Cycloheximide - - + + - - - -
Puromycin - + - + - + + +
CR-1-31-B Incubation Time 24 h 24 h 48 h 72 h
Ponceau S
SH-SY5Y
B
Vehicle Control + + - - - - - -
CR-1-31-B - - - - + + + +
Cycloheximide - - + + - - - -
Puromycin - + - + - + + +
CR-1-31-B Incubation Time 24 h 24 h 48 h 72 h
Ponceau S
Kelly
