# Supplementary material for: Eukaryotic Translation Initiation Factor 4AI: A Potential Novel Target in Neuroblastoma
Source: Cells. 2021 Feb 2;10(2):301. doi: 10.3390/cells10020301 (PMC7912938; doi:10.3390/cells10020301)
Supplement: Supplementary file 1 [file cells-10-00301-s001.zip › Supplementary_cells-1052933/SupplementaryFigure2Caption_cells-1052933.docx]

**Supplementary Figure 2:** **Puromycin Labelling of SH-SY5Y and Kelly Cells Upon CR-1-31-B Treatment.**
(**A**) Representative immunoblot and Ponceau S staining (loading control) for puromycin labelling in SH-SY5Y cells treated with vehicle control (VC), 20 nM CR-1-31-B for 24 h, 48 h and 72 h or cycloheximide [50 µg/ml]. Prior to harvest, cells were treated with puromycin dihydrochloride.
(**B**) Representative immunoblot and Ponceau S staining (loading control) for puromycin labelling in Kelly cells treated with vehicle control (VC), 4 nM CR-1-31-B for 24 h, 48 h and 72 h or cycloheximide [50 µg/ml]. Prior to harvest, cells were treated with puromycin dihydrochloride.
