# Supplementary figures and images for: Eukaryotic Translation Initiation Factor 4AI: A Potential Novel Target in Neuroblastoma
Source: Cells. 2021 Feb 2;10(2):301. doi: 10.3390/cells10020301 (PMC7912938; doi:10.3390/cells10020301)

A

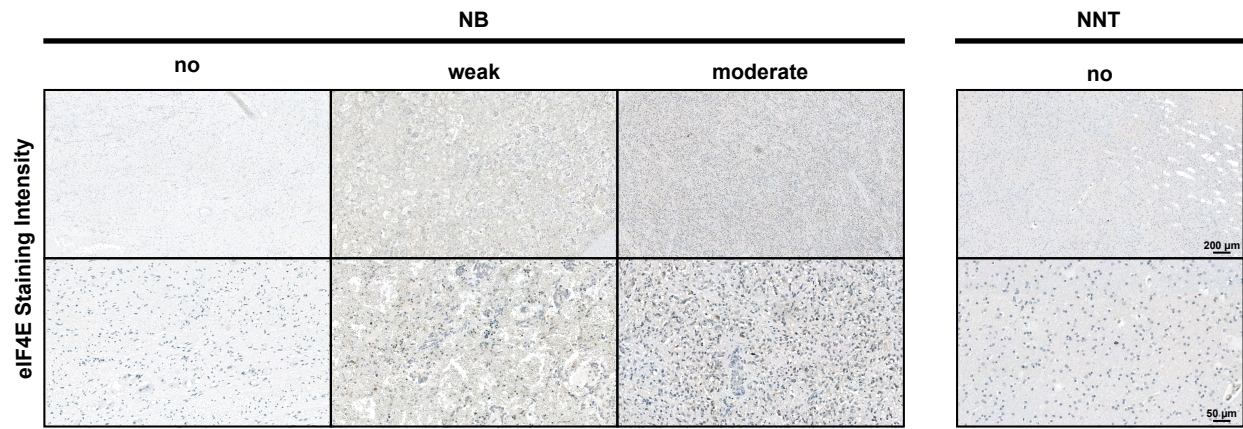

B

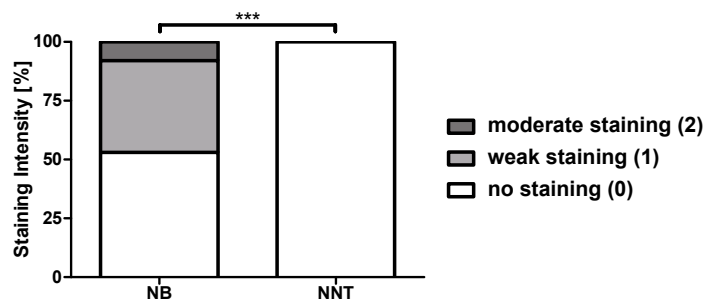

C

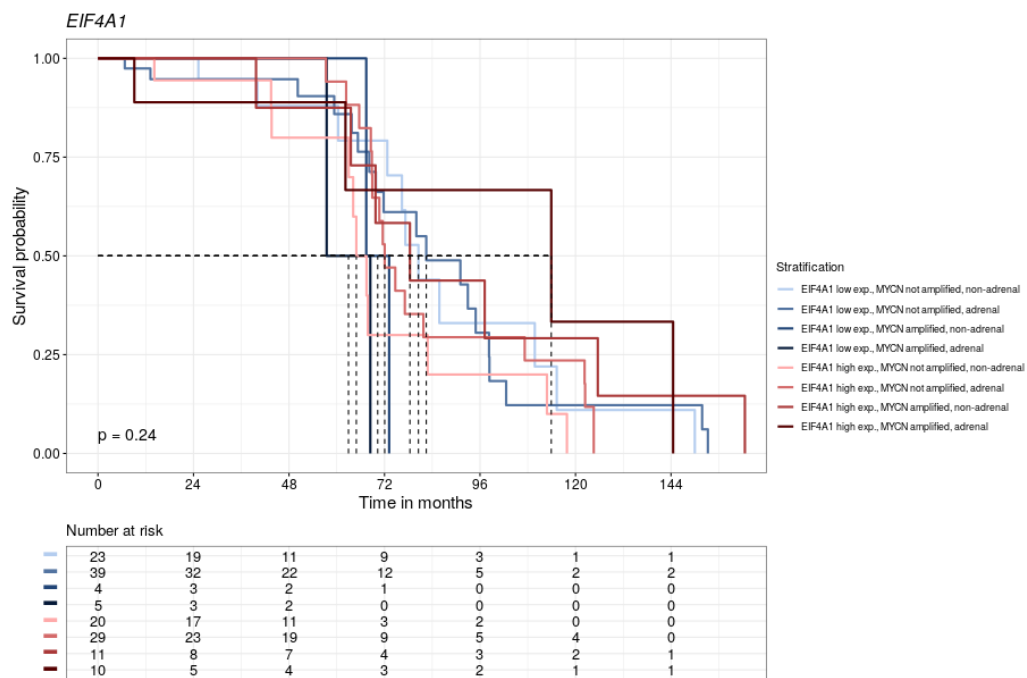

Supplement: Supplementary file 1 [file cells-10-00301-s001.zip › Supplementary_cells-1052933/SupplementaryFigure 1_cells-1052933.pdf]
